# Case Series on the Combined Therapy with Elexacaftor/Tezacaftor/Ivacaftor During Pregnancy in Women with Severe Cystic Fibrosis: A Retrospective Report from an Italian Centre

**DOI:** 10.3390/jcm14186520

**Published:** 2025-09-16

**Authors:** Paola Iacotucci, Jolanda Somma, Lorenza Ferrillo, Assunta Celardo, Valeria Conti, Costantino Di Carlo, Giuseppe Rengo, Graziamaria Corbi, Vincenzo Carnovale

**Affiliations:** 1 Department of Clinical Medicine and Surgery, University of Naples Federico II, 80131 Naples, Italy; paola.iacotucci@unina.it; 2 Department of Translational Medical Sciences, University of Naples Federico II, 80131 Naples, Italy; jolanda.somma@gmail.com (J.S.); lorenza.ferrillo@unina.it (L.F.); assunta.celardo2@unina.it (A.C.); giuseppe.rengo@unina.it (G.R.); vincenzo.carnovale@unina.it (V.C.); 3 Department of Medicine, Surgery and Dentistry Scuola Medica Salernitana, University of Salerno, 84081 Baronissi, Italy; vconti@unisa.it; 4 Department of Public Health, University of Naples Federico II, 80131 Naples, Italy; costantino.dicarlo@unina.it

**Keywords:** cystic fibrosis, CFTR modulator safety, pregnancy

## Abstract

In patients with cystic fibrosis (CF), infertility is a common issue in men, while women often experience subfertility. The introduction of CF transmembrane conductance regulator (CFTR) modulators has improved disease progression and enhanced quality of life, consequently leading to an increase in unplanned pregnancies. This article describes six cases of pregnancies in five patients diagnosed with severe CF who were treated with the combined therapy of elexacaftor/tezacaftor/ivacaftor (ETI). All women were under regular clinical and instrumental monitoring at the Regional CF Center for Adults at the University of Naples Federico II. The reported pregnancies were spontaneous, and all patients were followed throughout their pregnancies. Two pregnancies were carried to term by the same patient. All five patients with a severe CF phenotype were able to experience pregnancy without stopping their ETI treatment without any complications. In two cases, the patients chose to continue ETI therapy while breastfeeding, and there were no adverse events reported. A cesarean delivery was preferred in all cases to prevent potential respiratory distress. These five patients represent some of the few cases in Italy where pregnancy was achieved without interrupting treatment with ETI. However, the lack of more reliable data necessitates that doctors and patients carefully evaluate the risks and benefits of continuing or discontinuing treatment with CFTR modulators. In conclusion, the increasing number of pregnancies and the desire for children expressed by women with CF highlight the need for more data on the long-term effects of CFTR modulators.

## 1. Introduction

Cystic fibrosis (CF) is the most common autosomal recessive genetic disorder, affecting approximately 89,000 people worldwide [1]. It is caused by a variant in the CF Transmembrane Regulator (CFTR) gene, which encodes a protein located on the epithelial cell membrane that regulates chloride transport. The defect in this functional protein also disrupts sodium and water transport, resulting in increased volume and viscosity of secretions in various organs and systems. The main clinical manifestations associated with CFTR dysfunction primarily affect the respiratory system. These include recurrent respiratory infections, the development of bronchiectasis, and respiratory failure. Additionally, CF can lead to malabsorption in the gastrointestinal system, pancreatic insufficiency, and diabetes [2].

Infertility is another frequent CF manifestation. In men, infertility is correlated to congenital bilateral absence of vas deferens [3], which occurs in about 98% of CF patients [4]. In women, the sub-fertility rate is about 35%, higher than the 5–15% of the general population [5]. The causes of female subfertility are still to be defined, but since the female anatomy does not change, defects in the CFTR protein impair cervical mucus pH and viscosity [6].

In recent years, the improvement of therapies and the clinical introduction of CFTR modulators have significantly enhanced the course of the disease progression and quality of life.

Among the CFTR modulators, only the combination therapy elexacaftor/tezacaftor/ivacaftor (ETI) has increased the fertility rate [7] and the number of unplanned pregnancies [8]. The mechanisms underlying this phenomenon are unknown but are likely related to decreased cervical mucus density and/or improved pH, respiratory function, and nutritional status. A survey conducted in several CF centers in Europe, the UK, the US, Australia, and Israel found that CFTR modulators are generally well-tolerated during pregnancy and lactation, and their discontinuation leads to a worsening of the mother′s clinical condition [9]. However, the safety profile of these drugs remains uncertain. Recently, a few studies on animals have shown that exposure to CFTR modulators could affect fetal development, while data in humans are still limited [10,11,12].

## 2. Case Studies

This retrospective study describes all consecutive pregnancies in women on ETI at the Regional Cystic Fibrosis Center for Adults in Naples, Italy. Specifically, it reports on six pregnancies of five patients who underwent ETI combination therapy, detailing their severe CF before, during, and after delivery. A snapshot of the newborns’ conditions at birth was also provided. The CF severity was determined by using the Classes of CFTR mutations [13]. All 5 patients were classified in I and II classes, resulting in a diagnosis of severe CF.

All five women had been diagnosed with CF in childhood based on specific pathological sweat chloride levels (chloride > 60 mEq/L) and two CFTR mutations. The recruited patients received regular clinical and instrumental care at the Regional CF Center for Adults (Department of Translational Medical Sciences, University of Naples Federico II) and were followed through all their pregnancies. During pregnancy, a clinical standardized protocol is usually followed by the Center, consisting of medical examinations, blood tests, and an ultrasound every 1 month, in accordance with the gynecologist, and spirometry every 3 months. For this study, the data on the children were directly provided by the mothers.

Clinical, spirometry, and laboratory data, as well as information about the complications of enrolled patients, were extracted from local health records from January to June 2024.

The pregnancies described were spontaneous. ETI combination therapy consisted of the elexacaftor 100 mg/tezacaftor 50 mg/ivacaftor 75 mg administration in the morning, and ivacaftor 150 mg in the evening.

Two of these pregnancies were carried out by the same patient and are described as cases 1A and 1B. The women were initially informed of the lack of data on the safety of ETI therapy during pregnancy; however, given the high benefit of ETI therapy, they voluntarily decided to continue it. The study was conducted according to the guidelines laid down in the Helsinki Declaration and its amendments and was approved by the Ethics Committee of the University Federico II of Naples (Ref. 27/202). The protocol complied with the CARE guidelines for case reports [14] (see CARE checklist uploaded). All participants provided written informed consent. The description of each case follows.

### 2.1. Case 1A

Patient 1 was diagnosed with CF at the age of 40 days due to respiratory and intestinal symptoms. The disease was diagnosed by a pathological result of the sweat test and confirmed by molecular testing, which identified the mutations F508del and W1282X. This genetic combination characterizes a severe CF phenotype. The patient suffers from chronic bronchiectasis lung disease with airway colonization by Staphylococcus aureus and Pseudomonas aeruginosa, pancreatic insufficiency, CF-related diabetes (CFRD), gallstones, and osteopenia. Despite repeated attempts, she was unable to conceive for 12 months, so she opted for artificial insemination. She started treatment with ETI for compassionate use in December 2019, when her respiratory function was impaired with an FEV1 of around 33% as shown by spirometry. Several respiratory exacerbations occurred annually (average 4 exacerbations/year). The anthropometric parameters included a weight of 50.9 kg and a height of 1.60 m, resulting in a BMI of 19.88 kg/m^2^ (Table 1).

The therapy dramatically improved the patient’s health status one year after starting ETI treatment, with a 94% increase in FEV1 (FEV1 64%) and a reduction in the number of exacerbations (from 4 to 1 exacerbation/year). Weight and BMI also improved (Table 2). No adverse events occurred. Both liver and kidney functions remained normal during treatment.

The first spontaneous pregnancy, following the previous one induced by artificial insemination, occurred at the age of 38, about one and a half years after the start of ETI therapy. This was also the first pregnancy by the beginning of ETI therapy. The patient weighed 59 kg, had a BMI of 23.04 kg/m^2^, and an FEV1 of 61%. The pregnancy was regular. The patient did not discontinue ETI treatment. During pregnancy, she experienced renal colic with stone expulsion and required only oral antibiotic therapy for cystitis. There was no deterioration in respiratory function. At the end of the pregnancy, spirometry revealed an FEV1 of 66%. The mother was breastfeeding without discontinuing therapy, and no adverse events were recorded. The neonate weighed 2750 kg at birth and was in good clinical condition (Table 3), with a negative result for cystic fibrosis screening.

### 2.2. Case 1B

Almost 2 years after the first pregnancy, Patient 1 announced a second pregnancy. At this time, the patient weighed 53.8 kg, had a BMI of 21.01 kg/m^2^, and had an FEV1 of 63% (Table 2). Oral antibiotic therapy was also required during the second pregnancy, due to cystitis, but no respiratory exacerbation occurred. She was also diagnosed with hypothyroidism during pregnancy, which required replacement therapy (levothyroxine 50 mcg). Again, no deterioration in respiratory function was observed, with FEV1 remaining stable even after delivery (FEV1 62% three months postpartum). The pregnancy ended at 39 weeks’ gestation. The patient breastfed without discontinuing therapy, and no adverse events were recorded.

The baby weighed 3.570 kg, was healthy, and tested negative on neonatal CF screening (Table 3).

### 2.3. Case 2

Patient 2 is currently 41 years old. She was diagnosed with CF at the age of 1 year when she displayed respiratory and intestinal symptoms. The diagnosis was made based on the pathological results of 2 sweat tests and subsequently confirmed by molecular testing involving DNA extraction and mutation analysis of the CFTR protein. The mutations found, F508del/N1303K, defined a severe CF phenotype. She suffers from chronic bronchiectasis lung disease with airway colonization by Staphylococcus aureus, pancreatic insufficiency, gallstones, and CFRD. In 2008, she was diagnosed with Wolff-Parkinson-White syndrome, which was treated with transcatheter ablation through an abnormal double pathway of the radiofrequency Kent type at the left lateral and posterior sites. In November 2019, her respiratory function was severely impaired, with an FEV1 of about 31% prompting her to begin ETI therapy for compassionate use. Anthropometric characteristics included a weight of 53.3 kg, height of 1.65 m, and BMI of 19.58 kg/m^2^. She experienced an average of 5 respiratory exacerbations per year, some of which required hospitalization. Despite repeated attempts, she was unable to conceive for several years.

Treatment with ETI had no side effects and improved her respiratory function; her FEV1 was 45% after approximately 1 year of treatment. The therapy reduced the number of exacerbations to an average of 2 exacerbations per year (Table 1). Her pregnancy occurred after three years of ETI treatment at the age of 40. Spirometry revealed an FEV1 of 46%, her weight was 56 kg, and her BMI was 20.56 kg/m^2^. During pregnancy, hospitalization was necessary for severe hemoptysis, which required embolization. Despite this, no deterioration in her respiratory function was noted, and there were no complications for her or the fetus. Spirometry indicated an FEV1 of 45% two months after delivery (Table 2). The pregnancy lasted 36 weeks of gestation (Table 3). The newborn who had low weight (2.1 kg) required a short stay in the neonatal intensive care unit but made a full recovery. Genetic screening revealed that the newborn was a carrier of the F508del allele without CF (Table 3).

### 2.4. Case 3

Patient 3 is 27 years old. The diagnosis of CF was suspected at the age of 4 months due to respiratory and intestinal symptoms and subsequently confirmed by genetic testing: F508del/F508del, which defines a severe CF phenotype. The patient has chronic respiratory colonization by Staphylococcus aureus and Pseudomonas aeruginosa, pancreatic insufficiency, chronic sinusitis, previously surgically treated nasal polyposis, and allergic bronchopulmonary aspergillosis (ABPA) treated with Itraconazole. The patient has never attempted to become pregnant. Modulator therapy started in 2017 with Lumacaftor/Ivacaftor (LUM/IVA) and was replaced by ETI after about four years when her spirometry showed an FEV1 of 89%. The anthropometric parameters were weight 56 kg, height 1.61 m, and BMI 21.60 kg/m^2^. The patient had about two episodes of exacerbations per year (Table 1). The therapy improved the woman’s health status with a change in FEV1, which was 101% one year after the start of therapy. Weight and BMI also improved (weight 60.1 kg, BMI 22.19 kg/m^2^ after one year of therapy). The treatment did not cause any adverse events. Pregnancy occurred about two years after the start of treatment, at the age of 26. Spirometry revealed 101% FEV1, weight was 60.1 kg, and BMI was 23.18 kg/m^2^. A respiratory exacerbation required two oral cycles of antibiotic therapy but no hospitalization. Respiratory function did not change with an FEV1 that remained stable (FEV1 101% five months after delivery). The pregnancy ended in the 40th week of gestation. The baby girl weighed 3.100 kg, was healthy, and tested negative for neonatal screening for CF (Table 2).

### 2.5. Case 4

Patient 4 is currently 33 years old. The suspicion was raised at the age of 3 months after recurrent bronchopneumonia and gastrointestinal disturbances had occurred. The results of the pathological sweat test and subsequent molecular examination with DNA extraction and analysis of CFTR protein mutations confirmed the diagnosis. The mutation found, F508del/F508del, defined a severe CF phenotype. The patient has chronic bronchiectasis with colonization by Staphylococcus aureus and Pseudomonas aeruginosa, pancreatic insufficiency, and CFRD. Despite repeated attempts to conceive, she had not been pregnant for several years. She began modulator therapy with LUM/IVA in 2019 and switched to ETI in December 2020. The patient had significantly impaired respiratory function with an FEV1 of 29%. Respiratory exacerbations were approximately 3 per year. Anthropometric characteristics were weight 50.1 kg, height 1.50 m, and BMI 22.27 kg/m^2^. The therapy improved lung function by increasing the percent FEV1, which was 49% after one year of treatment. Weight increased to 56.6 kg, and BMI rose to 25.16 kg/m^2^. Pregnancy began after two years of therapy at the age of 31 years. The pregnancy was uneventful. The patient required two courses of oral antibiotics for respiratory exacerbations. The patient’s FEV1 was 50% at the end of the pregnancy. The baby was born in the 37th week of pregnancy. The baby girl weighed 3.270 kg at birth. There were no complications. The girl tested negative for CF (Table 2).

### 2.6. Case 5

Patient 5 was diagnosed with CF when she was ten years old. Her CF mutations are two F508del, which define a severe CF phenotype. The patient has chronic bronchopneumopathy with colonization by Pseudomonas aeruginosa and Staphylococcus aureus, pancreatic insufficiency, gallstones, and nasal polyposis. Despite repeated attempts, she was unable to conceive for 12 months, so she opted for artificial insemination. Treatment with ETI started in December 2021, when her FEV1 was 62%. Anthropometric parameters were weight 44 kg, height 1.52 m, and BMI 19.04 kg/m^2^ (Table 1). The first spontaneous pregnancy, following the previous one induced by artificial insemination, occurred one year after the beginning of ETI treatment. The FEV1 was 70%, the weight was 52.5 kg, and the BMI was 22.72 kg/m^2^. No respiratory exacerbations occurred during the pregnancy. The woman’s respiratory function and state of health had not changed. The FEV1 value of this patient was 73% at the end of the pregnancy. The pregnancy ended after 38 weeks. The child weighed 2.500 kg at birth, was negative for CF on newborn screening, and is currently healthy (Table 3).

## 3. Discussion

This article reports on 6 cases of women with CF undergoing ETI therapy, all of which resulted in uncomplicated pregnancies and healthy infants. In all cases, the pregnancies were spontaneous, although patients with CF typically experience increased infertility.

In the literature, pregnancy in women with moderate to severe pulmonary manifestations is associated with an increased risk of preterm birth, and infants born to mothers with cystic fibrosis have a higher risk of congenital anomalies [7]. Although the causes are unknown, female infertility in CF is linked to the expression of CFTR on the epithelial cells of the cervix. The mutation leads to an increase in the density of cervical mucus, which reduces sperm’s ability to penetrate the cervical system [6]. Furthermore, the altered functionality of CFTR impairs bicarbonate transport, leading to a pH imbalance in the uterus, which negatively affects sperm capacitation and oocyte fertilization [6]. Women with CF may also have low levels of the anti-Mullerian hormone, a marker of ovarian reserve [15], which partly explains the reduced fertility in these patients.

In 2012, the first drug modulator, called ivacaftor, was approved. Subsequently, three combined CFTR protein modulators—lumacaftor/ivacaftor (LUM/IVA), tezacaftor/ivacaftor (TEZ/IVA), and elexacaftor/tezacaftor/ivacaftor (ETI)—became available for most CF patients [16]. In 2021, ETI was approved for patients with CF over the age of 12 years with either two or a single F508del allele. In Italy, ETI was approved earlier than in other parts of Europe and the USA, but with certain limitations.

The modulators improved the nutritional, metabolic, and respiratory status of CF patients [17,18], as well as their fertility. The mechanisms underlying these improvements are not known, but for fertility, it is likely due to the reduction in the density of cervical mucus and the improvement in pH. The effect of these changes is a more fertile environment [6]. It is no coincidence that the number of pregnancies in adults with CF has increased since these drugs were introduced to the market [19,20]. However, as there is limited data on the safety of treatment during pregnancy, concerns remain regarding fetal exposure to CFTR modulators. In the past, animal studies have shown no adverse effects on chromosomes, organogenesis, and fetal survival [7]. Nonetheless, animal studies on the toxicity of IVA have indicated the development of congenital cataracts. Therefore, a baseline and annual ophthalmologic examination is recommended for children using IVA or IVA-containing products [7]. In the literature, 3 cases of infants exposed to IVA during pregnancy or lactation who were diagnosed with congenital cataracts have been described, but no significant visual damage was found in these children [21]. More recent studies on rats have shown that fetal exposure to the drug can have effects on neuronal development [10,11,12].

There is also limited data on the safety of this drug during breastfeeding. Research has shown that both IVA and LUM are excreted in breast milk [7]. A more recent study has indicated that ETI can pass into breast milk and fetal plasma, where it remains stable [22]. A working group of experts from Europe, Australia, and New Zealand has concluded that these drugs are likely safe during breastfeeding [23]. Although the approval of CFTR modulator therapies typically restricts use during pregnancy, shared decision-making between the CF care team and the patient can allow for informed consent to continue off-label therapy [24].

In the international context, some literature reports cases of women who continued using CF modulators during pregnancy without any complications for themselves or their children [25,26]. In the Cystic Fibrosis Centre of Newcastle, 41 women opted to continue ETI in pregnancy, and none of the children had CF, congenital anomalies, or cataracts [27]. Another multicenter retrospective study included all CF patients who reported conception from 1 January 2010, to 30 June 2021. Of 307 pregnancies, 77 (25.08%) were on ETI therapy, and 37 were taking other modulators. There were no statistically significant differences in rates of spontaneous abortions, cesarean section deliveries, preterm birth, or low-birth-weight infants between the group with and without CFTR modulator exposure [28]. In our study, all patients received ETI treatment throughout their pregnancies, and two cases also continued the treatment during breastfeeding.

Recently, a case was reported of a woman monitored at the CF Regional Center in Abruzzo, Italy, who took ETI for almost the entire pregnancy without complications [21]. This is the first study conducted in an Italian CF center with a larger number of women continuing ETI during pregnancy. The MAYFLOWERS study, a prospective multicenter observational study lasting approximately 35 months, is currently underway. It will follow 285 pregnant women throughout their pregnancies and for two years thereafter to learn about the effects of CF modulators during this period [29].

In all cases described, cesarean delivery was selected to prevent respiratory distress in patients who already had impaired respiratory function, as indicated by their FEV1 levels. While vaginal delivery can be considered [30], cesarean sections are often more frequently performed for precautionary reasons concerning the mother [31] and because there are no contraindications to cesarean delivery. During labor and delivery, the variability in respiratory volumes and ventilation can significantly increase the risk of fatigue and respiratory failure [32]. Therefore, our center, in coordination with the gynecologists, recommends cesarean delivery for women with compromised respiratory function, similar to the severe CF phenotype seen in the patients enrolled in this study, who exhibited a reduction in FEV1. Regarding the follow-up of the children, the pediatricians were informed of the need to monitor liver function and perform ophthalmological examinations. No pathological conditions were observed either during pregnancy or in the early years of life.

However, our study has some limitations. One significant limitation is that newborns of mothers affected by CF were not subjected to genetic testing or sweat tests. However, as previously suggested by Reix et al. [33], because CF is an autosomal recessive disease, if the complete CFTR gene analysis for the father is negative, there is no need to test the child. The diagnosis of CF was excluded from neonatal screening. Of the six newborns, only one underwent the genetic test despite the negative results of the neonatal screening, as the father is a carrier of a CFTR mutation. This genetic screening revealed that the newborn is a carrier of the F508del allele but does not have CF (Table 2). A second limitation is the small sample size, although these represent the first case reports in Italy of pregnancies during ETI therapy. Finally, the information on children was obtained from informal accounts by the mothers, who have not reported any hepatic, ocular, or neurological complications. In fact, the children have been followed up by the Pediatric Department.

## 4. Conclusions

This article discusses several cases in Italy where pregnancies occurred without interrupting treatment with ETI, and no complications were reported. It also details two cases where ETI treatment continued during breastfeeding without any adverse events. This study adds to the existing data supporting the safety of treatment during pregnancy and breastfeeding. However, the lack of reliable data emphasizes the importance of evaluating the risks and benefits of either continuing or discontinuing treatment with modulators. It′s essential to consider the potential risks associated with stopping modulators for women′s health, as well as the risks of fetal exposure to the drug. Given the increasing number of pregnancies and the growing desire among women with cystic fibrosis to have children, there is a pressing need for more comprehensive data on the effects of long-term modulator use.

## Figures and Tables

**Table 1:** Patients’ characteristics related to CF disease and the ETI therapy.

Case	Genotype	N° Exacerbations		ETI Start Date	FEV_1_		BMI (kg/m^2^)		ADRs by ETI
		Before ETI	After 1 Year on ETI		Before ETI	After 1 Year on ETI	Before ETI	After 1 Year on ETI	
1 (A & B)	F508del/W1282X	4	1	12/2019	33%	64%	19.88	22.14	none
2	F508del/N1303K	5	2	12/2019	31%	45%	19.58	20.56	none
3	F508del/F508del	2 *	1	12/2021	89% *	101%	21.60	22.19	none
4	F508del/F508del	3 *	1	12/2020	29% *	49%	22.27	25.16	none
5	F508del/F508del	2	1	12/2021	62%	70%	19.04	22.72	none

CF, Cystic Fibrosis; ETI, elexacaftor/tezacaftor/ivacaftor; FEV1, Forced Expiratory Volume in the first second; ADRs, Adverse Drug Reactions; CFRD, Cystic Fibrosis-related Diabetes. * In treatment with Lumacaftor/Ivacaftor.

**Table 2:** Patients’ characteristics related to the pregnancy and delivery period.

Case	Age at Pregnancy (yso)	Pregnancy Duration (Weeks)	BMI (kg/m^2^)		FEV_1_ (%)	
			Pre-Pregnancy	Post-Pregnancy	Pre-Pregnancy	Post-Pregnancy
1A	38	38	23.04	21.09	61	66
1B	40	39	21.01	20.97	63	62
2	40	36	20.56	22.18	46	45
3	26	40	23.18	23.84	101	101
4	31	37	25.16	25.11	49	50
5	40	38	22.72	19.52	70	73

yso, years old; BMI, Body Mass Index; FEV1, Forced Expiratory Volume in the first second; CF, Cystic Fibrosis.

**Table 3:** Some features of the newborn at birth.

Case	Weight (kg)	Gender	CF Diagnosis
1A	2.75	Male	No
1B	3.57	Male	No
2	2.10	Female	No (F508del carrier)
3	3.10	Female	No
4	3.27	Female	No
5	3.10	Female	No

CF, Cystic Fibrosis.

## Data Availability

Data sharing does not apply to this article.

## Abbreviations

The following abbreviations are used in this manuscript:

CF	cystic fibrosis
CFTR	cystic fibrosis transmembrane conductance regulator
ETI	elexacaftor/tezacaftor/ivacaftor
